# A Metapopulation Approach to African Lion (*Panthera leo*) Conservation

**DOI:** 10.1371/journal.pone.0088081

**Published:** 2014-02-05

**Authors:** Stephanie Dolrenry, Jennifer Stenglein, Leela Hazzah, R. Scott Lutz, Laurence Frank

**Affiliations:** 1 Nelson Institute for Environmental Studies, University of Wisconsin, Madison, Wisconsin, United States of America; 2 Living With Lions, Mpala Research Centre, Nanyuki, Kenya; 3 Forest and Wildlife Ecology, University of Wisconsin, Madison, Wisconsin, United States of America; 4 Museum of Vertebrate Zoology, University of California, Berkeley, California, United States of America; University of Illinois at Urbana-Champaign, United States of America

## Abstract

Due to anthropogenic pressures, African lion (*Panthera leo*) populations in Kenya and Tanzania are increasingly limited to fragmented populations. Lions living on isolated habitat patches exist in a matrix of less-preferred habitat. A framework of habitat patches within a less-suitable matrix describes a metapopulation. Metapopulation analysis can provide insight into the dynamics of each population patch in reference to the system as a whole, and these analyses often guide conservation planning. We present the first metapopulation analysis of African lions. We use a spatially-realistic model to investigate how sex-biased dispersal abilities of lions affect patch occupancy and also examine whether human densities surrounding the remaining lion populations affect the metapopulation as a whole. Our results indicate that male lion dispersal ability strongly contributes to population connectivity while the lesser dispersal ability of females could be a limiting factor. When populations go extinct, recolonization will not occur if distances between patches exceed female dispersal ability or if females are not able to survive moving across the matrix. This has profound implications for the overall metapopulation; the female models showed an intrinsic extinction rate from five-fold to a hundred-fold higher than the male models. Patch isolation is a consideration for even the largest lion populations. As lion populations continue to decline and with local extinctions occurring, female dispersal ability and the proximity to the nearest lion population are serious considerations for the recolonization of individual populations and for broader conservation efforts.

## Introduction

African lions (*Panthera leo*) once roamed the greater part of the African continent. As habitat generalists, they occupied a wide range of biomes with the exception of tropical rainforests and the interior of the Sahara desert [1]. Over the past century, it is estimated that lions’ range has been reduced by approximately 75% [2]. Across the majority of their present-day range, lion populations are now primarily associated with protected areas and managed hunting areas throughout sub-Saharan Africa [3].

Several strongholds of free-roaming lions remain in the East African countries of Tanzania and Kenya, home to more than half of the remaining lion population in Africa [2]. Lions are under threat in both countries, even though both countries have a strong tourism sector and Tanzania a strong trophy hunting sector, all based largely on lions [4], [5]. Lions are declining primarily due to indiscriminate killing by humans [6], [7], depletion of their prey base [8], [9], and overexploitation due to poor management of trophy hunting [5], [10]. Habitat conversion outside of protected areas has led to increasingly fragmented lion populations that are currently under threat of further isolation [11].

Although lions subsist in a wide variety of habitats, they are most successful in areas with low to medium human densities [12], [13], [14]. Due to anthropogenic pressures, the once nearly continuous network of lion populations across East Africa now exhibits a metapopulation structure: distinct populations within a wider landscape with limited migration between them [15], [16]. Monitoring of East African lion populations has shown that there is limited dispersal between the populations (i.e. observed dispersal between Tsavo West, Chyulu Hills and Amboseli National Parks and Namanga Forest Reserve, Kenya as well as between Serengeti National Park, Ngorongoro Conservation Area, Tanzania and the Masai Mara Reserve, Kenya [17], [18], [19]. Furthermore, lion populations have been observed to go extinct and recolonize (e.g. Amboseli National Park in the late 1990s [20], Sibiloi National Park (J. Harris, *pers. comm.*)) or to be ‘rescued’ by the immigration of new individuals after a population crash [19]. Although broader scale range maps show lions as continuous across much of East Africa, finer scale country-wide maps of known breeding or permanent lion populations reveal a distinct metapopulation pattern [2], [21], [22], [23]. Conservation planning for lions across East Africa requires an understanding of this network of, and exchange between, lion populations (i.e., the metapopulation).

Metapopulation analysis is an effective tool to better understand broad area population dynamics and the effects of species-specific life-history traits on population connectivity [24]. Current wildlife conservation and management benefit from a metapopulation approach because numerous wildlife populations are becoming increasingly isolated with regional extinction imminent for many species [25]. A metapopulation functions at a larger scale than individual populations and can provide further insight into the dynamics of each component population in reference to the system as a whole [26], [27].

Migrating individuals can re-colonize suitable patches after a local extinction has occurred, as well as enable a “rescue effect” in which immigrating individuals protect a dwindling local population from extinction [28], [29]. Successful recolonization is dependent on the dispersing individuals surviving ‘the matrix’, an area between populations which is generally not suitable for long-term survival and reproduction [30], [31], [32]. Traversing the matrix poses the largest threat for dispersing migrants, especially for large carnivores that are inherently in conflict with humans [30], [33], [34], [35].

Understanding the broader population dynamics of large carnivores can be particularly challenging given their longevity, large home ranges, and the lack of empirical data on dispersal abilities, particularly dispersal between habitat patches [30], [36]. Reliable knowledge of the species’ dispersal ability enables conservationists to maintain viable populations within proximity to other populations, thus ensuring sustainable exchange of individuals [37]. Although dispersal is one of the most important ecological processes, it remains one of the least understood [38]. Particularly with current rates of fragmentation and isolation, it is increasingly important to understand species’ dispersal characteristics and their role in maintaining the larger metapopulation [25].

As with other polygynous mammalian species [39], male lions nearly always disperse from their natal area, often traveling two to three times farther than females [18], [40], [41]. Females generally stay with their natal pride or establish neighboring home ranges ([18], ‘stepping stone’ dispersal – [42]). Based on these generally accepted dispersal characteristics, we combine empirical data on the life-history traits and current geographic status of lion populations in a fragmented landscape with a spatially realistic modeling approach [29], [43], [44] to examine the lion metapopulation of Kenya and Tanzania.

We use an incidence function model (IFM) to explore the probability of occurrence of particular lion populations (i.e. patches) within a broader lion metapopulation, where both distance between patches and area of patches vary [43]. We use the IFM to consider the effect of sex-specific dispersal characteristics on metapopulation connectivity, the impact of human densities, and the threat of isolation on the remaining lion populations [45] across Kenya and Tanzania.

## Materials and Methods

### Modeling Approach

Reliable models are insightful for conservation management as they can help managers evaluate the current state and consider a system’s future [44]. The simple and applicable incidence function model (IFM; [43], [46]) has high conservation value for evaluation of populations on a broad-scale [43]. The IFM was created to use empirical data to examine the effects of patch area and isolation on patch occupancy and is one of the most commonly used metapopulation models. An IFM can be built with snapshot presence/absence data of a species at a particular site, the simplest form of data that can be collected during field studies.

Incidence function models assume that suitable habitat occurs in discrete patches surrounded by unsuitable matrix, and that occupancy of each patch is determined by local colonization and extinction events [29], [45]. Extinction is negatively associated with patch area and colonization is negatively associated with patch isolation, so patch occupancy should increase with area and decrease with isolation [47]. Patch extinction and colonization are also assumed to depend on factors such as patch area (a proxy for local population size), spatial arrangement of patches, patch edge characteristics, dispersal ability of the species, and regional environmental stochasticity. In IFM models, the incidence is given as a function of connectivity and patch size [43]; in this study, factors such as lion occupancy and male and female dispersal distances, were calculated from empirical data on lion populations as well as verified metadata from various sources. Given the relatively high number of overall patches and the fraction of occupied patches during the short ‘snapshot’ period, we assumed the metapopulation was at equilibrium and thus executed the IFM [43].

To fit the IFM, we used a special case of generalized linear models with binomial error and logistic link function on the response of incidence (i.e. rate of occurrence) data for each habitat patch [48]. The necessary data inputs were incidence data (*J_i_*; 1 for presence, 0 for absence), area of each patch (*A_i_*), and coordinates of the center of each patch. We calculated all pairwise distances between patches (*d_ij_*), a dispersal parameter (α), estimated from actual dispersal data equal to 1/average dispersal as well as 1/maximum dispersal, and a connectivity value (*S_i_*). Connectivity (*S_i_*) is a function of occupancies *p_i_*, patch sizes *A_i_*, patch distances *d_ij_* and the species specific dispersion length parameter α. We used the negative exponential functional form of *S_i_* as suggested by Hanski [43].
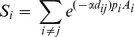



Due to impacts of human density on lion populations [13], [14], we included human density as a covariate in the incidence function models. Given the intense research on lion populations across Kenya and Tanzania as well as the high detectability of a breeding lion population when present (e.g. obvious tracks, roaring, livestock depredation, etc.), we believe the occurrence of false negatives (Type II error) to be negligible [15], [49], [50]. As discussed in Riggio et al. [2], the use of expert opinions and regional surveys for data of lion populations, particularly across East Africa, is likely to result in an inflation of lion range, not an underestimation.

### Data for the Model

**Figure 1:**
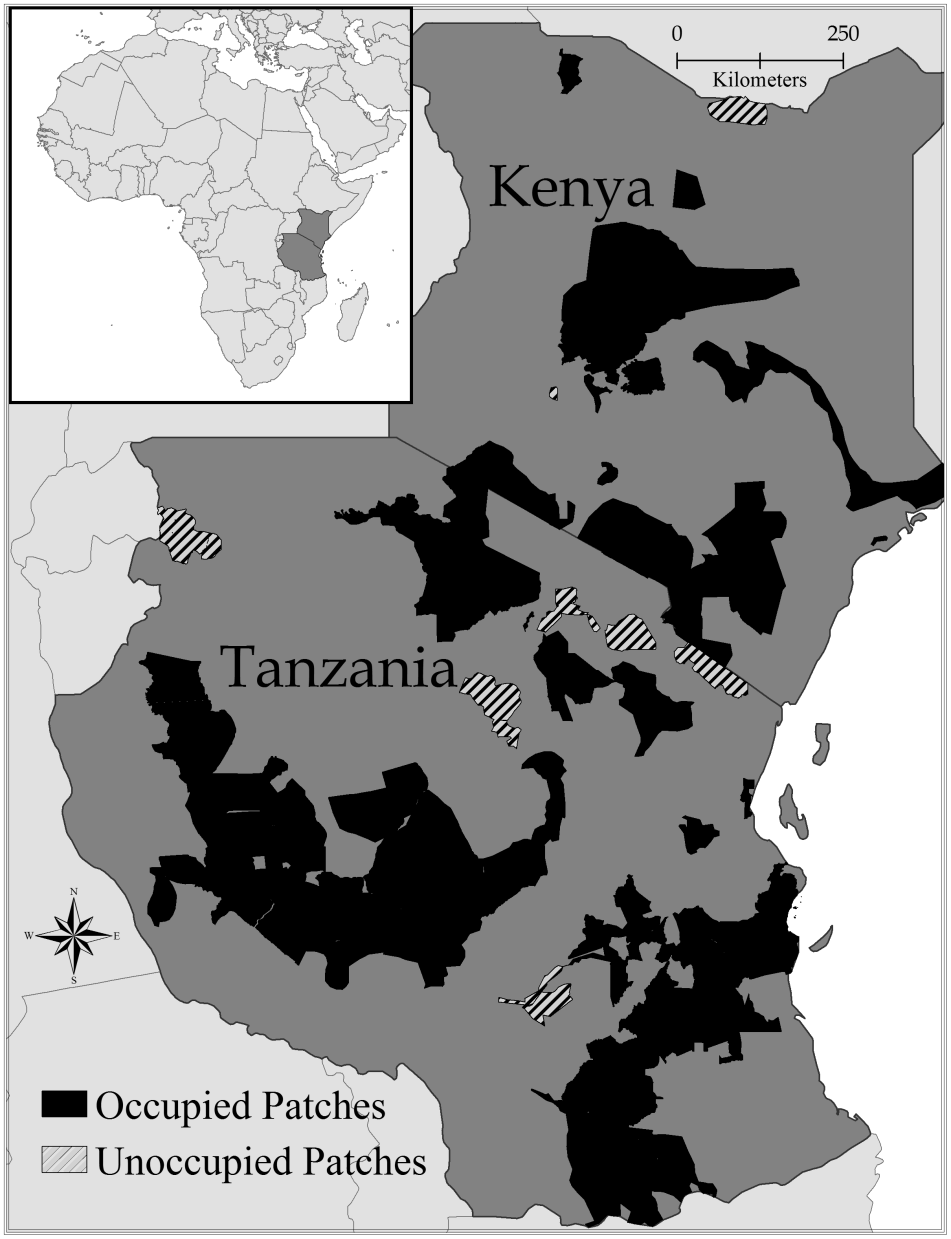
Map of study area in Africa. Darkened areas indicate patches of permanent lion populations (n = 25) across Kenya and Tanzania; black areas were considered occupied and striped areas were deemed unoccupied at time of survey.

We used the most recent and authoritative sources to identify and map known suitable habitat patches for lion populations from both countries, Mésochina et al. [22] for Tanzania and the Conservation and Management Strategy for Lions, Kenya (Kenya Wildlife Service [21]). In the Mésochina et al. [22] report, lion populations were identified by reported frequency of observation; weekly and monthly sightings were considered permanent populations, and we omitted areas for which there was no information available. In the Kenya report, we mapped all populations labeled as ‘known permanent’. Henceforth, we use the term ‘patch’ as synonymous with a distinct patch that was known to be suitable to support a lion population. In total, there were 25 patches with areas ranging from 86 to 127,515 km^2^ (Fig. 1).

Once all patches were identified, we sent maps to regional experts (conservationists, biologists, etc.) who were familiar with current lion population status in their areas. Experts identified areas that, to the best of their knowledge, contained a breeding lion population in December 2011-January 2012; they did not include areas where only male lions or dispersers were seen infrequently. This method gave a current presence-absence scenario (i.e. the ‘snapshot’) for patch occupancy.

We mapped and measured (km^2^) patches using ArcMap 10.1 [51], and used the centroid of each patch to calculate the pairwise Euclidean distances between patches (*d_ij_*). We subtracted the radii of the pair of patches for each pairwise distance, so the distances could be interpreted as the shortest straight-line distance between the edges of the paired patches. We gathered actual dispersal distances from the literature, unpublished reports, and the Lion Guardians program in the Amboseli ecosystem [17,40,41,Desert Lion [52], [53]. We used the maximum and average reported dispersal distances of 343 and 117 km for males and 128 and 50 km for females respectively as the dispersal parameter (α). We calculated human density for each patch from the 2009 census of Kenya (Kenya Bureau of Statistics [54]) at the sub-location level and the 2002 census for Tanzania (National Bureau of Statistics Tanzania [55]) at the ward level. We averaged the human density for each patch area.

### Statistical Methods

We followed the methods outlined in Hanski [43] and Oksanen [48] and performed the IFM in program R, version 2.14.0 [56]. We included the rescue effect in the model and added the mean human density for each of the patches as a covariate in the model. Therefore, our full model added one new term to Eq (9) in Oksanen [48]:
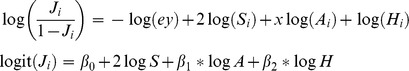
where *J_i_* is the incidence in patch *i*, *S_i_* is a measure of connectivity, *A*_i_ is each patch’s area, and *H_i_* is the mean human density of each patch. When fitting the full model, the intercept, 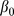 was log(*ey*), 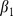 was an estimate of the stochasticity parameter 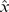, and 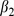 was the parameter estimate for human density entering the model as a linear factor.

Additional model parameters were: *A_0_*, the minimum suitable area, also referred to as critical patch area, and 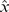, a stochasticity parameter. The critical patch area is the minimum patch size observed for occupancy [43]; it is the smallest area found to contain a lion population. When 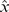 is large (>1), there is a range of patch sizes beyond which extinction becomes very unlikely, whereas if 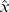 is small (<1), there is no such critical patch size and even large populations in large patches have a substantial risk of extinction. Lastly, we included the parameters *e*, the intrinsic extinction rate, and *y’*, the colonizing ability [43].

We used 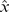 and *A_0_*, to find 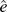:
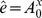



Then we were able to use 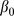 and 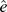 to solve for *y’* following Oksanen (2004):
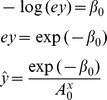



We used Akaike’s Information Criterion (AIC) to choose between nested models [57]. We ran male and female models independently, using average dispersal distance and maximum dispersal distance for each sex separately. The full models included human density additive to the patch area and the reduced models did not include human density. We used the connectivity term, 2ln(*S_i_*), as an offset in all eight models [48].

## Results

**Figure 2:**
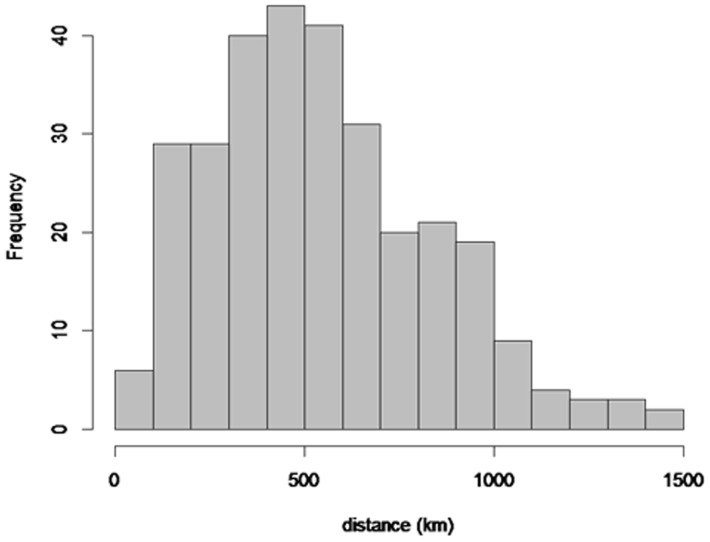
Pairwise distances between 25 Kenya and Tanzania lion population patches. Distance was calculated in kilometers, using data obtained during 2008–2010.

There were 25 total patches with an average area of 13,898 (SD 28,639) km^2^, and 60% of the patches were occupied (Fig.1). Mean human density value per patch was 162 (SD 475) per km^2^. The average distance among all patches was 546 km, varying from 63 to 1,461 kilometers (Fig. 2).

The male and female lion models without the human density were only marginally stronger than the models with human density included (Table 1). We found significant differences in connectivity values between the male and female models using both maximum dispersal distance (*t* = 21.16, *p<*0.001, df = 24) and average dispersal distances (*t* = 8.71, p<0.001, df = 24) as well as between the male models (avg male to max male *t* = 20.38, p<0.001, df = 24) and female models (avg female to max female t = 9.08, p<0.001, df = 24). As was expected from the model inputs, the greater ability of males to move through the matrix (male max *a = *0.0029, avg *a = *0.0086 versus female max *a = *0.0078, avg *a = *0.02), gave the male models reliably higher connectivity (Table 1). Three patches (3, 8, and 9; Fig. S1) had the lowest connectivity values across all models (Table S1). Particularly for the two largest patches (patches 3 and 8 with areas >40,000 km^2^; Fig. S1), the difference between the connectivity values of the two sexes was apparent (male max: mean (SD) = 14.78 (1.32), male avg = 0.72 (0.45); female max: mean (SD) = 1.02 (0.56), female avg = 0.01 (0.01); n = 2).

**Table 1:** Model outputs of eight African lion incidence function models.

	Mean log (HumanDensity) (SE)	ResidualDeviance		ResidualDF	AIC	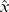 (SE)	*e*	*y'*	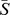 (SD)
Max Male (−H)	NA		35.08	23	39.08	0.44 (0.25)	7.04	3002.46	33.06 (10.52)
Max Male (+H)	−0.06 (0.24)		35.03	22	41.03	0.44 (0.24)	7.05	2381.55	33.06 (10.52)
Max Female (−H)	NA		41.61	23	45.61	0.82 (0.28)	38.40	470.13	8.53 (5.03)
Max Female (+H)	−0.11(0.25)		41.43	22	47.43	0.82 (0.28)	38.96	303.44	8.53 (5.03)
Avg Male (−H)	NA		42.95	23	46.95	0.88 (0.29)	51.14	397.48	7.92 (4.5)
Avg Male (+H)	−0.30 (0.30)		41.96	22	47.96	0.91 (0.30)	58.43	143.70	7.92 (4.5)
Avg Female (−H)	NA		69.99	23	73.99	1.83 (0.41)	3491.25	79.08	1.19 (1.08)
Avg Female (+H)	−0.40(0.41)		68.94	22	74.94	1.95 (0.49)	6008.43	23.57	1.19 (1.08)

Male and female models were calculated separately as they used different alpha values (male and female maximum (Max) and average (Avg) observed dispersal distances). Additional models were calculated with (+) and without (−) human density (H) as a covariate, added to both male and female models using both average and maximum dispersal distances. To discern model fit, human density coefficient with standard error (SE), residual deviance, residual degree of freedom and Akaike’s Information Criteria (AIC) are reported for each model. Model parameters reported: 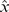 a stochasticity parameter reported with standard error; *e,* the intrinsic extinction rate; *y’*, the colonizing ability; and a measure of connectivity 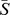, reported as an average for each model with standard deviation.

**Figure 3:**
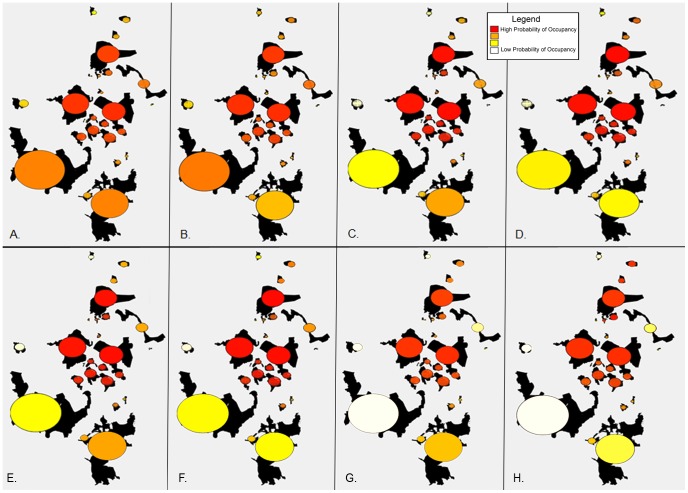
Probability of lion occurrence in identified lion population patches. Occurrence was calculated on 25 habitat patches in Kenya and Tanzania based on the incidence function model for A) male dispersal using maximum observed distance, without human density, B) male dispersal using maximum observed distance with human density, C) female dispersal using maximum observed distance without human density, D) female dispersal using maximum observed distance with human density model, E) male dispersal using average of observed distances, without human density, F) male dispersal using average of observed distances with human density, G) female dispersal using average of observed distances without human density, H) female dispersal using average of observed distances with human density model. In plotting the results, we used color to show the predicted incidence with warmer colors (redder) showing higher probability of incidence and yellow to white showing lower probability of incidence.

We used the estimated model parameters (Table 1) to determine probability of occupancy of the individual patches (Fig. 3). With regards to area size and its effect on patch occupancy, moderate-sized patches (2,000< area <40,000 km^2^) all had a relatively high probability of incidence (patch incidence *J_i_*: mean (SD) = 0.74 (0.26), n = 15), while the smaller patches (area ≤2,000 km^2^) had lower probabilities of lion occurrence (*J_i_*: mean (SD) = 0.40 (0.24), n = 8). Surprisingly, the largest patches (area ≥40,000 km^2^) also had much lower occurrence probabilities (*J_i_*: mean (SD) = 0.36 (0.21), n = 2). Across all scenarios, female models had a lower mean probability of patch incidence (max null models: mean (SD) = male 0.60 (0.18), female 0.57 (0.30); max human density models: male 0.55 (0.18), female 0.48 (0.30); avg null models: male 0.56 (0.31), female 0.43 (0.40); avg human density models: male 0.39 (0.31), female 0.25 (0.41)).

Adding the human density parameter did not influence either male or female models; the influence of human density in the reduced models was not significant (*p* = 0.81 and 0.32 for male models and 0.67 and 0.33 for the female models, using maximum and average dispersal distances). However, the human density parameter did decrease colonization for both sexes (colonization parameter, *C*; male max models *t* = −2.82, *p = *0.007, male avg models *t* = −4.49, *p* = <0.0001; female max models *t* = −2.91, *p = *0.005, female avg models *t* = −3.32, *p = *0.0017, at alpha level 0.05).

Furthermore, the patches with the highest associated human density (>2,000 people/km^2^, n = 2), had a decreased probability of occurrence in the models with human density added (mean* = *0.60 to 0.55 in male max models; 0.57 to 0.39 in male avg models and 0.57 to 0.48 in female max models; 0.43 to 0.25 in female avg models, Fig. 3).

## Discussion

The present-day fragmentation of the East African lion population into patches fits the metapopulation framework. Population patches are well delimited and cover only a fraction of the total area, allowing us to apply a metapopulation approach to the network of remaining lion populations across Kenya and Tanzania. In particular, we considered the impact of sex-specific dispersal characteristics on regional lion populations. With only one snapshot of population occupancy, strong differences were apparent between male and female dispersal abilities on connectivity and probabilities of occurrence.

Male lions’ ability to ‘rescue’ declining populations over 300 km away was strong, particularly since dispersal ability is pliant [58], [59]. If occupied patches, i.e. lion populations, are maintained and migration is allowed, then the males’ strong rescue ability allowed for higher probabilities of patch occupancy and stronger values of connectivity [60]. Lion survival while in the matrix and a constant flow of male dispersers from stable populations are important. If male lions are not able to disperse from stable populations, as may be the case where adult male survival is low, i.e., sport hunting areas [5], [61], [62], [63], this could result in a lower rescue effect for the broader metapopulation, causing an increased risk of extinction for local populations [28].

Fragmentation and isolation are among the key challenges to metapopulation maintenance [25], [64], [65]. Our results suggest that even the largest patches were negatively affected by isolation as shown by the lower occurrence probabilities and the low connectivity values observed for the two largest patches (3 and 8, Fig. S1). These areas are known to hold the majority of the remaining lions found in East Africa [2], [22]. However, because the IFM approach does not consider population numbers, the results are based solely on considerations of patch area and proximity to other patches.

Based on the model outputs, the higher probabilities of occurrence for the patches of northern Tanzania and southern Kenya (Fig. 3 and Table S1), concur with other studies [27] that a centrally located network of moderate-sized populations has greater probabilities of occurrence than either the few large isolated populations or the smaller well-connected patches. When examining a network of lion populations, considerations of patch isolation and fragmentation are vitally important, in conjunction with local-level considerations of individual population size and viability.

To avoid local extinction due to genetic isolation, it is necessary for a minimum of one or more dispersers in each generation to survive the matrix and recolonize other patches, allowing populations to persist in stochastic environments [27], [30], [31], [66], [67], [68], [69]. The models showed males to have poor ‘colonizing ability’ (*y’*) in comparison to females; this ‘colonizing ability’ is independent of the ability to move across the matrix (*S_i_*) [43]. Our *y’* estimates suggest that even though male lions can move across the matrix better than female lions (increased ability to disperse), when compared to females, they have a harder time establishing themselves in a patch. This makes sense biologically since males are more nomadic and move territories frequently whereas females disperse less often and shorter distances than males. Females choose to generally stay within their natal range or establish neighboring home ranges; once they reach a suitable patch, they tend to stay there [18], [40], [70].

Once patch extinction has occurred, recolonization would be dependent upon female lions, less capable dispersers, being able to reach the empty patch. Since female lion dispersal ability is limited, when populations go extinct, recolonization cannot occur if distances between patches exceed female dispersal ability or if females are not able to survive crossing the matrix. This has profound implications for the overall metapopulation of lions; the female models showed an intrinsic extinction rate from five-fold to a hundred-fold higher than the male models (Table 1).

Dispersal occurs in and is dependent upon the matrix, in this case largely non-protected, human-dominated landscapes [32]. Little is known about lion dispersal and survival in human-dominated areas. We suggest conservation efforts for lions in East Africa focus on increasing lion survival while crossing the matrix to ensure connectivity on a broad-scale. Additionally, conservation efforts could aim to improve accuracy of regional lion presence/absence data and the knowledge of occurrence and status of breeding lion populations through the establishment of a lion sightings database, as is done with other species (e.g. avian sightings databases [71], [72]). Observational data on lions across East Africa would increase our understanding of the distribution of lions and provide a foundation for more effective lion conservation.

As top predators, lions are especially vulnerable and even minor human-induced changes (either negative or positive) can have large impacts [73], [74], [75]. Wildlife or stakeholder carrying capacity (i.e. the amount of wildife local residents are willing to ‘live with’ - [76], [77]) is a concept that aims to increase tolerance of local communities, as a step toward increasing the number or density of lions that communities are able to tolerate or accept. Traditional-based participatory monitoring programs have been shown to increase local tolerance of lions [78], thus allowing for greater connectivity.

Improving human tolerance to allow movement of even a few individuals through non-protected or partially protected areas could play a vital role in conservation of carnivores, both to maintain populations and to allow greater exchange between them [78], [79]. Even though protected areas are necessary for long-term persistence [73], alone they are not enough as most of the lands essential for supporting carnivore dispersal and thus population connectivity, are outside protected areas, in lands that are affected by humans [1], [80].

In addition to suitable habitat and available prey, human tolerance for carnivores can create corridors between larger stable unfenced populations. Packer et al. [81] found fenced lion populations to occur at higher densities, but fences inhibit all exchange between populations, leading to further ecosystem fragmentation, loss of dispersal and migration routes, genetic isolation as well as reduced conservation value of wildlife in matrix areas [82]. If lions are seen as a benefit by the human communities living in the matrix, they will tolerate sharing the landscape with a challenging species allowing greater population viability, increased economic opportunities for the communities and better conservation.

Even though human density was not a significant factor for either model, we suspect that further study (i.e. subsequent surveys over longer time periods) would reveal longer-term impacts of human density on extinction and/or colonization rates. However, greater community tolerance and adaptive management policies may be more important factors than human population densities [35], [79], [83]. We also found the stochasticity parameter (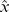) to be less than one for all models, implying that in this metapopulation, no lion population is immune from going extinct [84], further suggesting the need and urgency for conservation action on a broad geographic scale.

Incidence function models allowed a simple initial evaluation of lion populations and their connectivity across Kenya and Tanzania. Using only data on areas, locations and patch occupancies, we were able to analyze the effects of patch area, isolation and dispersal abilities on lion populations on a metapopulation scale. More explicit examination of extinction and colonization rates of the patches as well as further exploration of factors affecting dispersal and survival in the matrix areas would provide additional insights for making effective policy decisions about large-scale lion populations across Africa. Extirpation of lions throughout Africa is an immediate concern and the metapopulation approach and the incorporation of life-history traits are essential tools for understanding the broader networks of populations and movements between them.

## Supporting Information

Figure S1 **Map of study area in Africa.** Darkened areas indicate patches of permanent lion populations (n = 25) across Kenya and Tanzania; black areas were considered occupied and striped areas were deemed unoccupied at time of survey. In this map, patches were numbered 1 to 25, corresponding with Patch ID of Table S1.
(TIF)

Table S1 **Model outputs from eight incidence function models, varying the dispersal distance used by either male or female, maximum observed (Max) or average of observed (Avg) as well as the incorporation of human density (Hum) as a covariate in the models.** Models without human density covariate are labeled as Null. Patches were given unique identification numbers 1 through 25. Each patch had an associated area (km^2^) and human density (number people per km^2^) variable. Outputs were: *S_i_* as an estimate of connectivity associated with both male and female, maximum and average dispersal distance models; *J_i_* as an estimate of patch occurrence, *E* estimates of patch extinction, and *C* estimate of patch colonization. All are given for the eight models and twenty-five patches.
(DOCX)
