# Supplementary figures and images for: A Metapopulation Approach to African Lion (Panthera leo) Conservation
Source: PLoS One. 2014 Feb 5;9(2):e88081. doi: 10.1371/journal.pone.0088081 (PMC3914926; doi:10.1371/journal.pone.0088081)

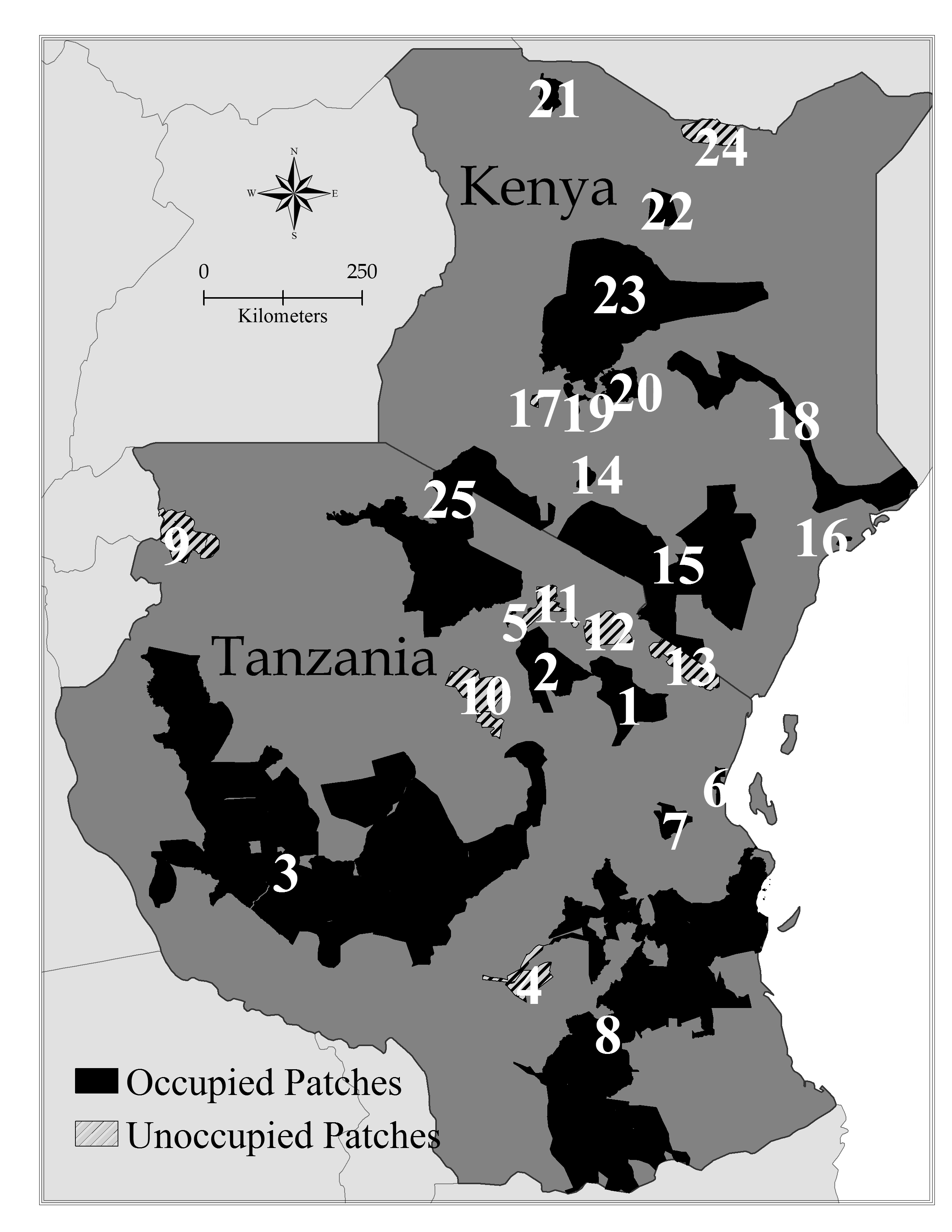

Supplement: Figure S1 — Map of study area in Africa. Darkened areas indicate patches of permanent lion populations (n = 25) across Kenya and Tanzania; black areas were considered occupied and striped areas were deemed unoccupied at time of survey. In this map, patches were numbered 1 to 25, corresponding with Patch ID of Table S1. (TIF) [file pone.0088081.s001.tif]
