# Supplementary material for: A Metapopulation Approach to African Lion (Panthera leo) Conservation
Source: PLoS One. 2014 Feb 5;9(2):e88081. doi: 10.1371/journal.pone.0088081 (PMC3914926; doi:10.1371/journal.pone.0088081)
Supplement: Table S1 — Model outputs from eight incidence function models, varying the dispersal distance used by either male or female, maximum observed (Max) or average of observed (Avg) as well as the incorporation of human density (Hum) as a covariate in the models. Models without human density covariate are labeled as Null. Patches were given unique identification numbers 1 through 25. Each patch had an associated area (km2) and human density (number people per km2) variable. Outputs were: Si as an estimate of connectivity associated with both male and female, maximum and average dispersal distance models; Ji as an estimate of patch occurrence, E estimates of patch extinction, and C estimate of patch colonization. All are given for the eight models and twenty-five patches. (DOCX) [file pone.0088081.s002.docx]

| **Patch ID** | **Area (km^2^)** | **Human Density** | **(S_i_) Max Male** | **(S_i_) Max Female** | **(S_i_) Avg Male** | **(S_i_) Avg Female** | **(J_i_) Max Male Null** | **(J_i_) Max Male Hum** | **(J_i_) Max Female Null** | **(J_i_) Max Female Human** | **(J_i_) Avg Male Null** | **(J_i_) Avg Male Hum** | **(J_i_) Avg Female Null** | **(J_i_) Avg Female Human** |
| --- | --- | --- | --- | --- | --- | --- | --- | --- | --- | --- | --- | --- | --- | --- |
| 1 | 7951 | 19.10 | 38.2950 | 10.3880 | 8.8272 | 0.9966 | 0.7802 | 0.7917 | 0.9034 | 0.9152 | 0.9144 | 0.9338 | 0.9804 | 0.9889 |
| 2 | 7297 | 19.22 | 39.7281 | 11.0034 | 9.4026 | 1.3954 | 0.7863 | 0.7974 | 0.9072 | 0.9186 | 0.9183 | 0.9366 | 0.9882 | 0.9932 |
| 3 | 127516 | 28.74 | 13.8493 | 0.6213 | 0.3989 | 0.0005 | 0.6103 | 0.6212 | 0.2451 | 0.2659 | 0.2020 | 0.2433 | 0.0024 | 0.0050 |
| 4 | 3080 | 32.55 | 24.4770 | 4.1631 | 3.3722 | 0.2545 | 0.4890 | 0.4985 | 0.4086 | 0.4291 | 0.4029 | 0.4249 | 0.3650 | 0.4231 |
| 5 | 86 | 45.98 | 43.1335 | 13.1429 | 11.4009 | 2.0375 | 0.3826 | 0.3866 | 0.2687 | 0.2760 | 0.2464 | 0.2250 | 0.0499 | 0.0363 |
| 6 | 645 | 17.22 | 31.6192 | 8.1626 | 7.0617 | 1.5534 | 0.4460 | 0.4641 | 0.4247 | 0.4607 | 0.4265 | 0.4842 | 0.5500 | 0.6249 |
| 7 | 1910 | 23.22 | 31.3913 | 7.9212 | 6.8037 | 1.3640 | 0.5608 | 0.5747 | 0.6284 | 0.6556 | 0.6430 | 0.6825 | 0.8731 | 0.9047 |
| 8 | 71740 | 2401.90 | 15.7167 | 1.4139 | 1.0387 | 0.0161 | 0.6105 | 0.5615 | 0.5122 | 0.4238 | 0.5081 | 0.2574 | 0.4239 | 0.1960 |
| 9 | 4739 | 41.89 | 16.9302 | 0.9631 | 0.6524 | 0.0028 | 0.3561 | 0.3616 | 0.0500 | 0.0529 | 0.0356 | 0.0366 | 0.0002 | 0.0002 |
| 10 | 4852 | 116.05 | 41.3331 | 11.0818 | 9.4601 | 1.5019 | 0.7691 | 0.7631 | 0.8766 | 0.8714 | 0.8881 | 0.8579 | 0.9787 | 0.9738 |
| 11 | 2197 | 128.80 | 49.0452 | 17.4264 | 15.4124 | 3.4927 | 0.7682 | 0.7611 | 0.9018 | 0.8962 | 0.9127 | 0.8829 | 0.9831 | 0.9762 |
| 12 | 2992 | 96.92 | 48.9035 | 17.5149 | 15.5012 | 3.3017 | 0.7904 | 0.7866 | 0.9227 | 0.9206 | 0.9329 | 0.9167 | 0.9892 | 0.9869 |
| 13 | 3446 | 23.41 | 42.7310 | 12.7558 | 11.0222 | 1.7417 | 0.7539 | 0.7642 | 0.8767 | 0.8890 | 0.8884 | 0.9061 | 0.9706 | 0.9799 |
| 14 | 598 | 3443.77 | 42.8277 | 12.6689 | 10.8333 | 1.3284 | 0.5883 | 0.5331 | 0.6256 | 0.5261 | 0.6208 | 0.5183 | 0.4376 | 0.3031 |
| 15 | 27967 | 135.43 | 38.5239 | 9.3620 | 7.7552 | 0.5678 | 0.8617 | 0.8568 | 0.9551 | 0.9526 | 0.9616 | 0.9656 | 0.9939 | 0.9961 |
| 16 | 166 | 46.62 | 26.3199 | 2.8083 | 2.0586 | 0.0269 | 0.2353 | 0.2383 | 0.0279 | 0.0290 | 0.0187 | 0.0195 | 0.0000 | 0.0000 |
| 17 | 200 | 373.00 | 43.8032 | 14.3852 | 12.6280 | 2.8920 | 0.4805 | 0.4556 | 0.4677 | 0.4233 | 0.4581 | 0.3131 | 0.3316 | 0.1623 |
| 18 | 7000 | 226.80 | 28.7660 | 3.7877 | 2.8626 | 0.0505 | 0.6545 | 0.6383 | 0.5284 | 0.4993 | 0.5011 | 0.5639 | 0.0924 | 0.1476 |
| 19 | 692 | 232.27 | 40.3037 | 12.8883 | 11.2896 | 2.4917 | 0.5743 | 0.5563 | 0.6609 | 0.6321 | 0.6692 | 0.5987 | 0.7815 | 0.7028 |
| 20 | 2641 | 594.57 | 38.3713 | 12.1255 | 10.6238 | 2.2990 | 0.6874 | 0.6599 | 0.8379 | 0.8056 | 0.8540 | 0.7551 | 0.9725 | 0.9430 |
| 21 | 1500 | 3.11 | 16.9903 | 2.2339 | 1.7389 | 0.0574 | 0.2517 | 0.2850 | 0.0994 | 0.1329 | 0.0868 | 0.2290 | 0.0078 | 0.0377 |
| 22 | 2125 | 13.96 | 25.6158 | 6.0347 | 5.1039 | 0.6090 | 0.4711 | 0.4924 | 0.5172 | 0.5599 | 0.5269 | 0.6295 | 0.6252 | 0.7641 |
| 23 | 26468 | 61.83 | 31.1937 | 8.5872 | 7.3860 | 1.0595 | 0.7996 | 0.8000 | 0.9448 | 0.9460 | 0.9558 | 0.9687 | 0.9981 | 0.9991 |
| 24 | 2900 | 1.41 | 22.8472 | 4.8763 | 4.0854 | 0.4462 | 0.4482 | 0.5014 | 0.4743 | 0.5769 | 0.4843 | 0.7008 | 0.6127 | 0.8600 |
| 25 | 36743 | 26.50 | 33.8257 | 6.8667 | 5.5867 | 0.3829 | 0.8441 | 0.8506 | 0.9347 | 0.9413 | 0.9430 | 0.9500 | 0.9919 | 0.9951 |
|  |  |  |  |  |  |  |  |  |  |  |  |  |  |  |
| **Patch ID** | **Area (km^2^)** | **Human Density** | **(E) Max Male Null** | **(E) Max Male Hum** | **(E) Max Female Null** | **(E) Max Female Hum** | **(E) Avg Male Null** | **(E) Avg Male Hum** | **(E) Avg Female Null** | **(E) Avg Female Human** | **(C) Max Male Null** | **(C) Max Male Hum** | **(C) Max Female Null** | **(C) Max Female Hum** |
| 1 | 7951 | 19.10 | 0.1376 | 0.1371 | 0.0245 | 0.0238 | 0.0183 | 0.0140 | 0.0003 | 0.0001 | 0.0002 | 0.0003 | 0.0008 | 0.0019 |
| 2 | 7297 | 19.22 | 0.1429 | 0.1424 | 0.0263 | 0.0256 | 0.0198 | 0.0152 | 0.0003 | 0.0001 | 0.0001 | 0.0001 | 0.0000 | 0.0001 |
| 3 | 127516 | 28.74 | 0.0408 | 0.0406 | 0.0025 | 0.0024 | 0.0016 | 0.0011 | 0.0000 | 0.0000 | 0.0002 | 0.0003 | 0.0009 | 0.0022 |
| 4 | 3080 | 32.55 | 0.2085 | 0.2078 | 0.0534 | 0.0520 | 0.0424 | 0.0333 | 0.0014 | 0.0005 | 0.0002 | 0.0003 | 0.0007 | 0.0017 |
| 5 | 86 | 45.98 | 1.0000 | 0.9981 | 1.0000 | 0.9858 | 1.0000 | 0.8753 | 1.0000 | 0.5811 | 0.0001 | 0.0002 | 0.0003 | 0.0007 |
| 6 | 645 | 17.22 | 0.4136 | 0.4125 | 0.1920 | 0.1881 | 0.1687 | 0.1390 | 0.0250 | 0.0113 | 0.0002 | 0.0003 | 0.0008 | 0.0018 |
| 7 | 1910 | 23.22 | 0.2571 | 0.2562 | 0.0789 | 0.0770 | 0.0647 | 0.0516 | 0.0034 | 0.0014 | 0.0000 | 0.0001 | 0.0000 | 0.0001 |
| 8 | 71740 | 2401.90 | 0.0525 | 0.0522 | 0.0041 | 0.0039 | 0.0026 | 0.0019 | 0.0000 | 0.0000 | 0.0001 | 0.0002 | 0.0003 | 0.0007 |
| 9 | 4739 | 41.89 | 0.1726 | 0.1720 | 0.0375 | 0.0365 | 0.0290 | 0.0225 | 0.0006 | 0.0002 | 0.0001 | 0.0001 | 0.0002 | 0.0004 |
| 10 | 4852 | 116.05 | 0.1709 | 0.1702 | 0.0368 | 0.0358 | 0.0284 | 0.0220 | 0.0006 | 0.0002 | 0.0003 | 0.0004 | 0.0014 | 0.0033 |
| 11 | 2197 | 128.80 | 0.2418 | 0.2410 | 0.0704 | 0.0687 | 0.0571 | 0.0454 | 0.0026 | 0.0010 | 0.0002 | 0.0003 | 0.0007 | 0.0016 |
| 12 | 2992 | 96.92 | 0.2112 | 0.2105 | 0.0546 | 0.0533 | 0.0435 | 0.0342 | 0.0015 | 0.0006 | 0.0001 | 0.0001 | 0.0001 | 0.0003 |
| 13 | 3446 | 23.41 | 0.1985 | 0.1978 | 0.0487 | 0.0474 | 0.0384 | 0.0301 | 0.0012 | 0.0004 | 0.0003 | 0.0004 | 0.0014 | 0.0033 |
| 14 | 598 | 3443.77 | 0.4276 | 0.4264 | 0.2043 | 0.2001 | 0.1803 | 0.1489 | 0.0287 | 0.0132 | 0.0001 | 0.0001 | 0.0001 | 0.0002 |
| 15 | 27967 | 135.43 | 0.0793 | 0.0790 | 0.0088 | 0.0085 | 0.0060 | 0.0044 | 0.0000 | 0.0000 | 0.0002 | 0.0003 | 0.0007 | 0.0018 |
| 16 | 166 | 46.62 | 0.7497 | 0.7480 | 0.5836 | 0.5741 | 0.5594 | 0.4801 | 0.2999 | 0.1608 | 0.0000 | 0.0001 | 0.0000 | 0.0000 |
| 17 | 200 | 373.00 | 0.6909 | 0.6894 | 0.5010 | 0.4925 | 0.4745 | 0.4050 | 0.2132 | 0.1118 | 0.0002 | 0.0003 | 0.0006 | 0.0013 |
| 18 | 7000 | 226.80 | 0.1455 | 0.1450 | 0.0272 | 0.0265 | 0.0205 | 0.0158 | 0.0003 | 0.0001 | 0.0001 | 0.0001 | 0.0001 | 0.0002 |
| 19 | 692 | 232.27 | 0.4011 | 0.4000 | 0.1813 | 0.1775 | 0.1585 | 0.1304 | 0.0219 | 0.0099 | 0.0002 | 0.0003 | 0.0005 | 0.0013 |
| 20 | 2641 | 594.57 | 0.2230 | 0.2223 | 0.0605 | 0.0590 | 0.0486 | 0.0384 | 0.0019 | 0.0007 | 0.0002 | 0.0003 | 0.0005 | 0.0012 |
| 21 | 1500 | 3.11 | 0.2858 | 0.2849 | 0.0962 | 0.0940 | 0.0800 | 0.0643 | 0.0053 | 0.0022 | 0.0001 | 0.0002 | 0.0003 | 0.0008 |
| 22 | 2125 | 13.96 | 0.2453 | 0.2445 | 0.0723 | 0.0706 | 0.0588 | 0.0468 | 0.0028 | 0.0011 | 0.0002 | 0.0003 | 0.0004 | 0.0010 |
| 23 | 26468 | 61.83 | 0.0812 | 0.0809 | 0.0092 | 0.0089 | 0.0063 | 0.0047 | 0.0000 | 0.0000 | 0.0001 | 0.0002 | 0.0002 | 0.0005 |
| 24 | 2900 | 1.41 | 0.2141 | 0.2134 | 0.0561 | 0.0546 | 0.0447 | 0.0352 | 0.0016 | 0.0006 | 0.0000 | 0.0000 | 0.0000 | 0.0000 |
| 25 | 36743 | 26.50 | 0.0704 | 0.0701 | 0.0070 | 0.0068 | 0.0047 | 0.0035 | 0.0000 | 0.0000 | 0.0000 | 0.0000 | 0.0000 | 0.0000 |
